# Case Report: Sciatic nerve schwannoma - a rare cause of sciatica

**DOI:** 10.12688/f1000research.11108.1

**Published:** 2017-03-14

**Authors:** Sunil Munakomi, Pratyush Shrestha

**Affiliations:** 1 Department of Neurosurgery, Nobel Teaching Hospital, Biratnagar, Nepal; 2 National Institute of Neurosurgery and Allied Sciences, Kathmandu, Nepal

**Keywords:** sciatica, sciatic nerve, schwannoma

## Abstract

Herein we report a rare case of a sciatic nerve schwannoma causing sciatica in a 69-year-old female. Sciatic nerve schwannoma is a rare entity. It should always be considered as a possible cause of sciatica in patients that present with symptoms of sciatica with no prolapsed disc in the lumbar spine and a negative crossed straight leg raise test. Timely diagnosis and complete excision of the lesion leads to complete resolution of the symptoms of such patients.

## Introduction

Sciatic nerve schwannoma is a rare cause of sciatica ^1– 3^. However, it remains a probable diagnosis in patients that present with symptoms of sciatica with no prolapsed disc in the lumbar spine and a negative crossed straight leg raise test, suggesting the presence of a far lateral disc. Magnetic resonance imaging (MRI) along the course of the sciatic nerve is the cornerstone for coming to a correct diagnosis and thereafter implementing a right therapeutic decision ^1^. This case report highlights the need to consider sciatic nerve schwannoma as a possible cause of a sciatica in patients that have a negative lumbar spine MRI, so that the correct therapeutic decision can be made.

## Case report

**Figure 1.**
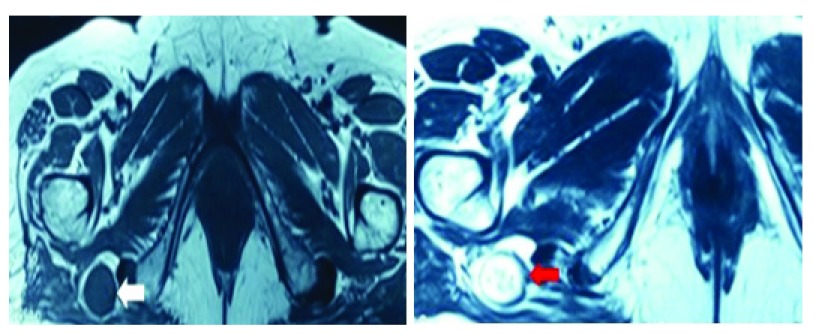
MRI scan showing an oval, well circumscribed and homogenous lesion lying near the ischial tuberosity.

**Figure 2.**
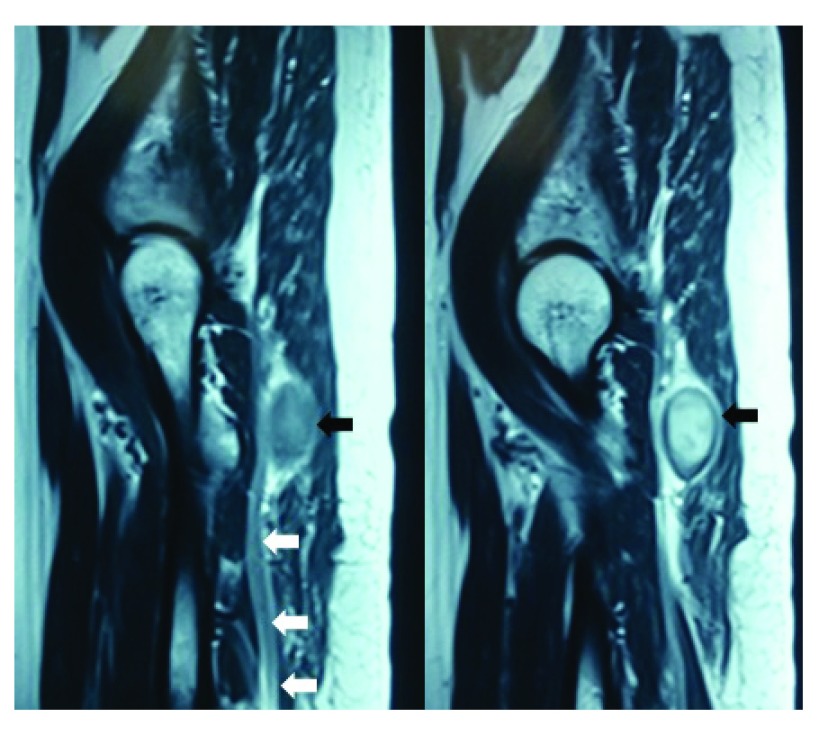
MRI scan revealing the unusual location of the tumor with a hypo-intense rim along the course of the sciatic nerve.

A 69-year-old female from eastern Nepal presented to our outpatient clinic with a history suggestive of right sided sciatica for the last 2 years. She had been evaluated before for the same, but without any positive diagnosis. The patient denied any history of trauma or any alteration in her bladder and bowel habits, or of any symptoms which is suggestive of intermittent claudication. Upon neurological examination, the power in all the muscle groups in her lower limbs was normal - 5/5 as per the MRC Muscle scale (used with the permission of the Medical Research Council). Her ankle and the knee reflexes were normal and she had no sensory indifference in any of the dermatomes in the affected limb, as compared to the normal limb. There was no wasting of the extensor digitorum brevis muscle. Straight leg raise test and a crossed straight leg raise test were both negative. Her stance was also normal. While sitting in a squatting position, the patient complained of an exaggeration of her symptoms. We thereafter made a differential diagnosis of either a sciatic nerve tumor or a Pyriformis syndrome. Radio imaging with help of an MRI scan revealed the presence of a sciatic tumor alongside the sciatic nerve, near the ischial tuberosity on the right side ( Figure 1). The unusual location of the lesion was in favor of a schwannoma rather than a neurofibroma ( Figure 2).

**Figure 3.**
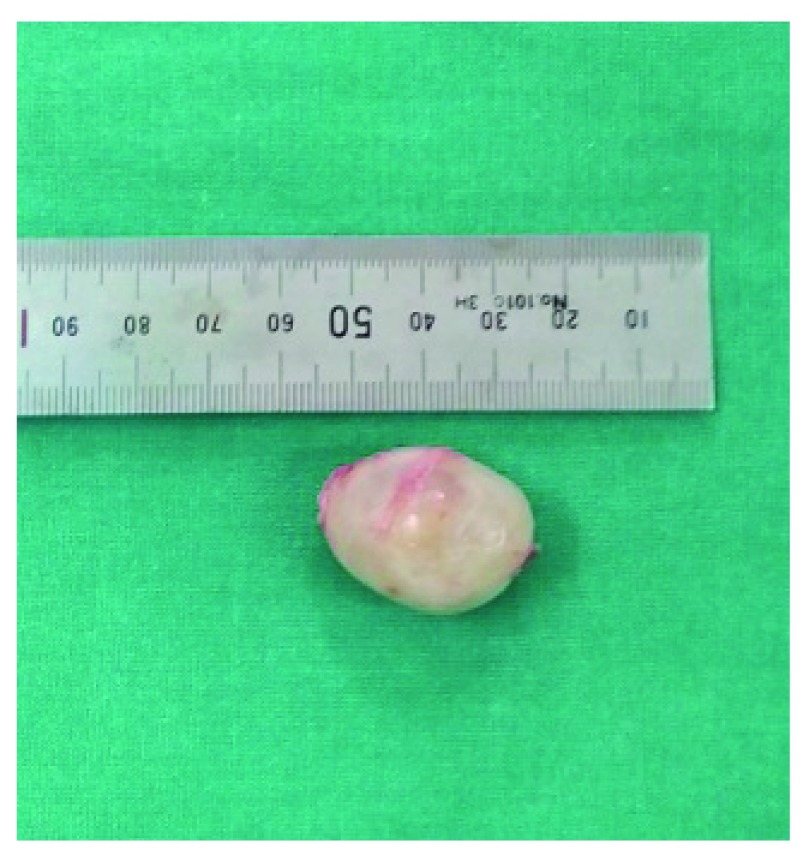
Gross image of the excised lesion.

The patient was counseled for the operative intervention that would remedy her persistent symptoms. A subgluteal approach was taken for the surgical corridor. Intra-operatively, a 3×3 cm ^2^ well circumscribed lesion was seen lying within the sciatic nerve. It was carefully dissected off the nerve fascicles and fully removed ( Figure 3). The sciatic nerve was confirmed to be intact intra-operatively with the aid of an intra-operative nerve monitor.

**Figure 4.**
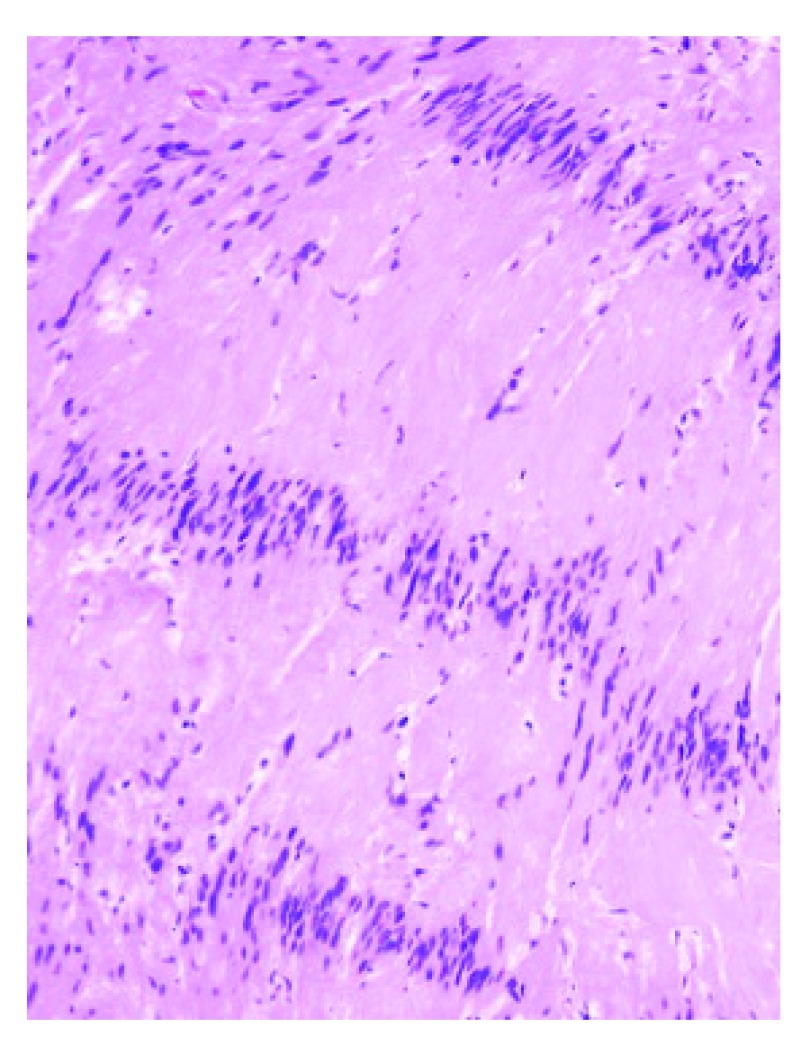
Photomicrograph of tissue taken from the lesion excised from the patient showing cells within Antoni A and Antoni B regions that are characteristic of schwannoma.

Postoperatively, the patient was completely free of her previous symptoms. She made a full recovery with no adverse events and was discharged on the fifth day. The histopathological report confirmed the diagnosis of a sciatic nerve schwannoma, owing to the presence of Antoni A and B areas and Verocay bodies ( Figure 4). The patient returned to her follow up visit at 1 month completely asymptomatic.

## Discussion

Sciatic nerve schwannoma is a rare cause of sciatica, occurring only in 1 of every 100 cases ^1^. It should be suspected in a patient who presents with a typical history of sciatica but with MRI scans that fail to reveal any inter-vertebral disc prolapse in the lumbar spine ^1^. Other differential diagnoses included sciatic nerve tumors, a far lateral disc or Pyriformis syndrome. The main imaging modality for the diagnosis of sciatic nerve schwannoma was MRI imaging of the affected sciatic nerve.

Neurofibromas are intrinsic lesions that cause fusiform dilatation of the nerve, since the lesions are intermixed with the nerve ^1^. On the other hand, schwannomas are placed in such a way that nerve fascicles are being pushed to the periphery, allowing their safe preservation following excision of the schwannoma ^2, 4– 6^. Intra-operative nerve monitoring helps immensely to outline the course of the nerve and define the boundary of the tumor during its removal. Definitive diagnosis however is only possible after the histopathological studies.

For the excision of such lesions, both a transgluteal or a subgluteal approach can be taken ^7, 8^. In both these approaches, the patient is placed in a prone position. The sciatic nerve invariably lies midway between the ischial tuberosity, medially, and the greater trochanter, laterally. A subgluteal approach may lead to prolonged discomfort due to retraction of the soft tissues and the gluteal muscles ^9^. A transgluteal approach may sometimes lead to disastrous consequences due to retraction of the muscle arteries within the pelvis. However it provides a wider surgical corridor up to the sciatic notch ^9^.

Histopathology is the mainstay for differentiating the type of tumor involved, with only an occasional need for immunohistochemical markers like S100 ^2, 5, 6^.

Recurrence is uncommon following complete excision ^6^. Malignant transformation of such lesion is rare ^1, 2^. Good outcome is expected following its complete excision because of its benign nature ^2^.

## Conclusion

Though rare, sciatic nerve schwannoma should be taken into account for the differential diagnosis in a patient presenting with long standing sciatica without positive findings of a disc in the lumbar spine. MRI imaging of the nerve is prudent for the diagnosis of the lesion. It is imperative to outline the course of the nerve and to define the boundary of the lesion to preserve the nerve fascicles. This can be facilitated with the aid of an intraoperative nerve monitor.

## Consent

Both written and verbal informed consent for publication of images and clinical data related to this case was sought and obtained from the patient.

## References

[1] Rhanim A El Zanati R Mahfoud M: A rare cause of chronic sciatic pain: Schwannoma of the sciatic nerve. *J Clin Orthop Trauma.* 2013;4(2):89–92. 10.1016/j.jcot.2013.04.001 26403631 PMC3880523

[2] Banshelkikar S Nistane P: Intrasubstance Schwannoma of Posterior Tibial Nerve Presenting as Lumbo-Sacral Radiculopathy. *J Orthop Case Rep.* 2015;5(2):35–37. 27299039 10.13107/jocr.2250-0685.268 PMC4722585

[3] Eroglu U Bozkurt M Ozates O: Sciatic nerve schwannoma: case report. *Turk Neurosurg.* 2014;24(1):120–2. 24535807 10.5137/1019-5149.JTN.7915-13.0

[4] Haspolat Y Ozkan FU Turkmen I: Sciatica due to Schwannoma at the Sciatic Notch. *Case Rep Orthop.* 2013;2013:510901. 10.1155/2013/510901 23762699 PMC3671531

[5] Chikkanna JK Gopal S Sampath D: Mystery of Sciatica Resolved - A Rare Case Report. *J Clin Diagn Res.* 2016;10(1):RD04–RD05. 10.7860/JCDR/2016/17865.7108 26894136 PMC4740664

[6] Kumar S Ralli M Sharma J: Sciatic schwannoma: A rare entity. *Clin Cancer Investig J.* 2015;4(6):720–2. 10.4103/2278-0513.169113

[7] Patil PG Friedman AH: Surgical exposure of the sciatic nerve in the gluteal region: anatomic and historical comparison of two approaches. *Neurosurgery.* 2005;56(1 Suppl):165–171; discussion 165–71. 10.1227/01.NEU.0000144169.84261.9D 15799806

[8] Socolovsky M Garategui L Campero A: Exposure of the sciatic nerve in the gluteal region without sectioning the gluteus maximus: an anatomical and microsurgical study. *Acta Neurochir Suppl.* 2011;108:233–240. 10.1007/978-3-211-99370-5_36 21107965

[9] Montano N Novello M D'Alessandris QG: Intrapelvic sciatic notch schwannoma: microsurgical excision using the infragluteal approach. *J Neurosurg.* 2013;119(3):751–5. 10.3171/2013.3.JNS121161 23581593

