# Supplementary material for: Case Report: Sciatic nerve schwannoma - a rare cause of sciatica
Source: F1000Res. 2017 Mar 14;6:267. [Version 1] doi: 10.12688/f1000research.11108.1 (PMC5365229; doi:10.12688/f1000research.11108.1)
Supplement: Peer review history

# Peer review: Referee response for version 1

**DOI:** 10.5256/f1000research.11983.r21114

**Published:** 2017-03-20

**Authors:** Guru Dutta Satyarthee

**Affiliations:** 1 Department of Neurosurgery, Neurosciences Centre, All India Institute of Medical Sciences (AIIMS), New Delhi, Delhi, India

Microsurgical excision of sciatic nerve schwannoma with good outcome

 The authors reported an interesting case of sciatic nerve  schwannoma in a 69-year old female, who was symptomatic for two-years, magnetic resonance imaging revealed  presence of a mass lesion causing expansion of  right sciatic nerve. A provisional diagnosis of peripheral nerve sheath tumor was made.  She underwent micro-surgical excision   using a sub-gluteal approach, intraoperative expansion of the sciatic nerve was observed, and nerve fascicles were  carefully separated from mass lesion  and  well circumscribed lesion was excised, with physiological nerve monitoring. Histopathology was suggestive schwannoma with amelioration of symptoms ^1^.

 Schwannoma   is a benign peripheral nerve tumor of   Schwann cells origin, that usually presents as a slow growing, solitary, well  circumscribed mass. The   sciatic nerve involvement represents less than 1% of all schwannoma ^2^.

 Peripheral nerve sheath  schwannoma symptom relates to alteration in the  function of  nerve and surrounding muscle and neurovascular bundles, and mostly commonly   present with  paraesthesia or pain  of insidious onset and progresses slowly ^2^^-^^4^. Pain is a much more common symptom than motor deficits.  Pain due to sciatic nerve schwannoma may simulate chronic sciatica pain produced due to a prolapse of lumbar herniated disc.  Physical examination may reveal the presence of a lump along the course of the sciatic nerve, which is tender, have mobility along the transverse axis but limited along the course of the nerve, and with a typical positive Tinel sign. However, pre-operative diagnosis  cannot reliably in most cases (even with magnetic resonance imaging) distinguish among  schwannoma, neurofibroma, or plexiform neurofibroma, but aids in delineating shape, size, location, extent and relation with parent nerve and adjacent neurovascular structures and muscle. Imaging plays a limited role in distinguishing among peripheral nerve sheath tumors. Magnetic resonance imaging may show presence of fusiform mass with characteristic  tapering cephalad and distal ends, fasciculation sign and  split fat signs ^3^^-^^4^. The mass is located eccentrically, well-circumscribed, and shows isointense signal on T1-weighted images and hyperintense signal and peripheral rim demonstrate hypo-intensity signal representing capsule T2 weighted images ^3^^-^^4^.

 The diagnoses of sciatic nerve  schwannoma depends on MRI of sciatic nerve carried out in the event of normal MRI findings of lumbar spine, but the patient still complains of the persistence of sciatica-like pain. Treatment of epineurium encapsulated tumour is microsurgical excision with careful preservation of the sciatic nerve fascicles. Histopathological examination of resected specimen confirms the definitive diagnosis ^1^^-^^5^.

 Kim et al. analysed 397 cases of peripheral nerve sheath tumor, out of which 91% were benign and the rest were malignant. A total of 251 were located in the brachial plexus region or upper limb. 141 benign lesions were related to brachial plexus tumors, and the rest (110) belonged to upper-extremity benign peripheral nerve sheath tumors. In contrast to upper limb, the peripheral nerve sheath tumor involving lower-limbs included 32 cases of schwannomas and 53 cases of neurofibroma ^5^.  

I have read this submission. I believe that I have an appropriate level of expertise to confirm that it is of an acceptable scientific standard.

## References

[1] MunakomiSShresthaP: Case Report: Sciatic nerve schwannoma - a rare cause of sciatica. *F1000Research*.2017;6: 10.12688/f1000research.11108.1 10.12688/f1000research.11108.1 28413620PMC5365229

[2] BlanchardCDam-HieuPZagnoliFBellardS: [Chronic sciatic pain caused by sciatic nerve schwannoma]. *Rev Med Interne*.2008;29(9) : 10.1016/j.revmed.2008.01.008 748-50 10.1016/j.revmed.2008.01.008 18325638

[3] LiCSHuangGSWuHDChenWTShihLSLiiJMDuhSJChenRCTuHYChanWP: Differentiation of soft tissue benign and malignant peripheral nerve sheath tumors with magnetic resonance imaging. *Clin Imaging*.32(2) : 10.1016/j.clinimag.2007.05.006 121-7 10.1016/j.clinimag.2007.05.006 18313576

[4] WasaJNishidaYTsukushiSShidoYSugiuraHNakashimaHIshiguroN: MRI features in the differentiation of malignant peripheral nerve sheath tumors and neurofibromas. *AJR Am J Roentgenol*.2010;194(6) : 10.2214/AJR.09.2724 1568-74 10.2214/AJR.09.2724 20489098

[5] KimDHMurovicJATielRLMoesGKlineDG: A series of 397 peripheral neural sheath tumors: 30-year experience at Louisiana State University Health Sciences Center. *J Neurosurg*.2005;102(2) : 10.3171/jns.2005.102.2.0246 246-55 10.3171/jns.2005.102.2.0246 15739552

# Peer review: Referee response for version 1

**DOI:** 10.5256/f1000research.11983.r20984

**Published:** 2017-03-15

**Authors:** Ravi Dadlani

**Affiliations:** 1 Department of Neurosurgery, Apollo Speciality Hospital, Nellore, Andhra Pradesh, India

I congratulate the authors on an interesting case report.

 It is a well written report but I would like to suggest a few additional points. 

 Since several such case reports have been published earlier, it would be interesting if the authors could add a 'review of literature'. A single tabulated format with some interesting characteristic, such as 

 the exact location of the tumor along the course of the sciatic nerve.

 It would also be interesting to see a small table with other 'sciatica mimicks'. Personally I have seen lumbosacral plexus tumors presenting with sciatica. 

 The article may be accepted for indexing with these minor additions.

I have read this submission. I believe that I have an appropriate level of expertise to confirm that it is of an acceptable scientific standard.
